# Supplementary material for: Association between oral microbiome and seven types of cancers in East Asian population: a two-sample Mendelian randomization analysis
Source: Front Mol Biosci. 2023 Nov 21;10:1327893. doi: 10.3389/fmolb.2023.1327893 (PMC10702768; doi:10.3389/fmolb.2023.1327893)
Supplement: Supplementary file 1 [file DataSheet1.ZIP › Supplementary Materials/MR plots of saliva/saliva═╝/Gastric cancer/pheno.2064_to_gastric cancer_forest.pdf]

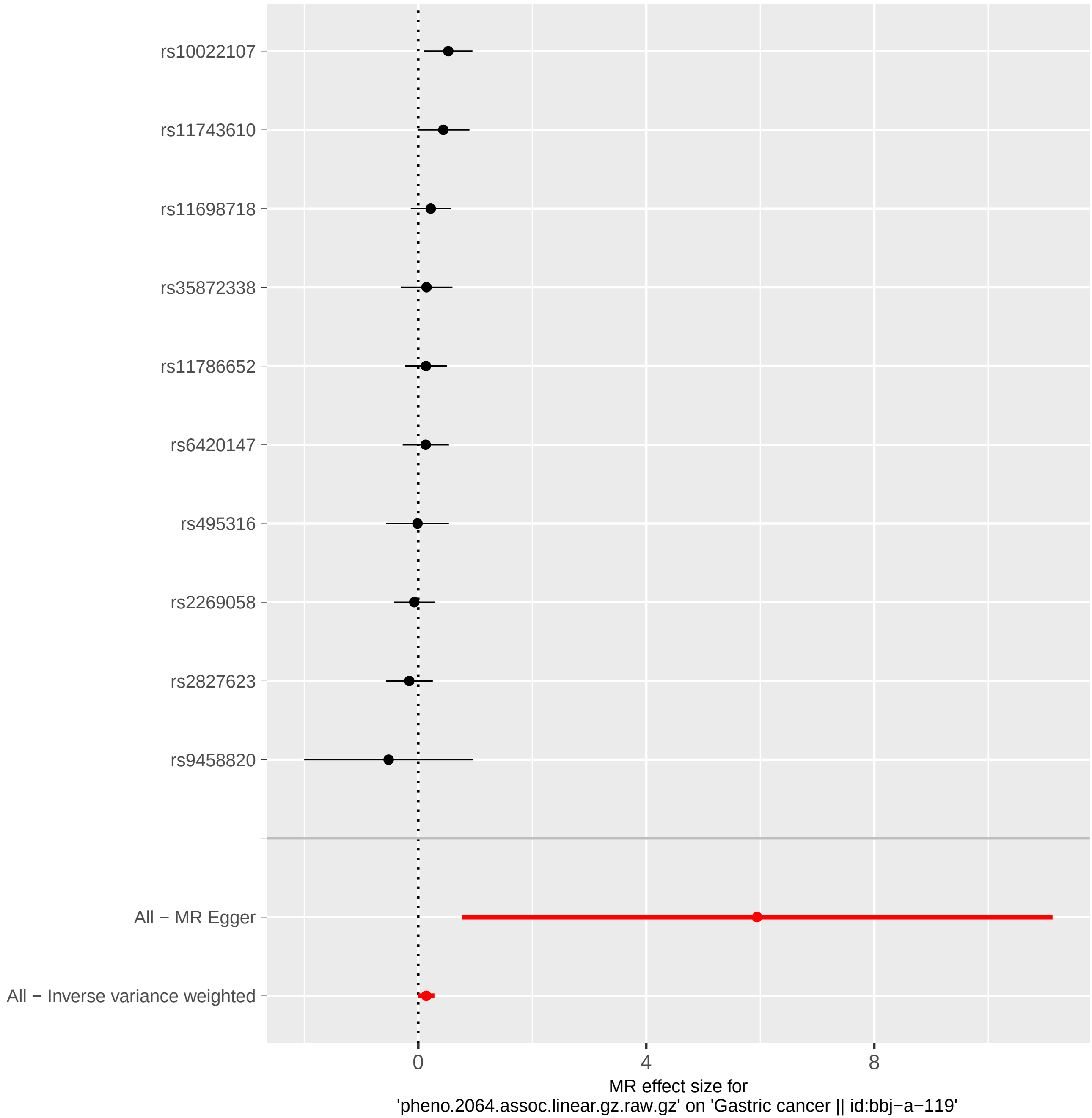

rs10022107
rs11743610
rs11698718
rs35872338
rs11786652
rs6420147
rs495316
rs2269058
rs2827623
rs9458820
All − MR Egger
All − Inverse variance weighted
0
4
8
MR effect size for
'pheno.2064.assoc.linear.gz.raw.gz' on 'Gastric cancer || id:bbj−a−119'
